# Supplementary material for: First evidence for an aposematic function of a very common color pattern in small insects
Source: PLoS One. 2021 Feb 11;16(2):e0237288. doi: 10.1371/journal.pone.0237288 (PMC7877781; doi:10.1371/journal.pone.0237288)
Supplement: S1 File — (DOCX) [file pone.0237288.s001.docx]

**S1 Appendix. Salticid rearing.**

Several cage designs were tested, but the most successful (in terms of preventing the escape of spiderlings, optimizing environmental conditions, and facilitating the filming of spider behavior) was one made of acrylic, 14 cm x 14 cm and 18 cm high, with two lateral circular areas (10 cm diameter) with small holes for aeration covered with an ultra-fine mesh. Including vegetation in the cages helps mitigate the effects of captivity [1], and we found that the best results were obtained by using the same plant species that harbored the egg sac in the field; water was provided in test tubes plugged with cotton. We occasionally observed advanced stage juveniles preying on younger stages, but this behavior was sufficiently uncommon that it was deemed unnecessary to separate specimens individually during the life cycle. However, cannibalism increased as the number of individuals per cage increased and therefore no more than 10 individuals were maintained in a single cage; they were generally separated during the fourth week after hatching, which is when they usually begin to disperse.

A survival rate of 53% was achieved by providing young spiderlings and juveniles (less than 200 days old) with whiteflies, *Bemisia tabaci* Gennadius, and then transitioning to *Drosophila melanogaster* Meigen; older spiders fed with *Drosophila* showed a survival rate of 94%. Whiteflies were reared on young eggplants (*Solanum melongena*) planted in pots inside aluminum cages that were hermetically closed with anti-aphid fabric. Weekly plant watering and room temperature near 24 º C were crucial. In some cases when populations of *B. tabaci* were low we used an undetermined species of *Aleyrodes* found on *Emilia fosbergii, Sonchus oleraceus* and *S. asper.* For *Drosophila* rearing, glass vials with autoclaved *Drosophila* diet (2 bananas, 50 g of oatmeal, 175 ml water and 1.75 ml of propionic acid) were used for rearing at room temperature protected from drafts and direct sunlight.

**S2 Appendix. Choice of oil paint for the lures.**

Six mixtures of different [water](http://www.cobra.royal-talens.com/) soluble, solvent-free oil paints (Gamblin®) for the black and orange colors were prepared and tested; the mixtures consisted of combinations between permanent orange, Van Dyke brown, ivory black and Venetian red. Their reflectance spectra were measured using a spectrophotometer (508 PV Craic) coupled to a microscope (Eclipse LV100ND, Nikon). A spectralon standard was used as a diffuse white reference and the experimental setup parameters were the same as in a previous study [2].

Two methods were used for choosing the paint mixture for the lures:

a) Calculating CIELAB color coordinates according to the CIE standard using the reflectance spectra, and then calculating the color differences. In general, color coordinates allow the geometrical representation of colors. In particular, the CIE color spaces are recognized as having important characteristics such as being device-independent and having a perceptual linearity [3]. The CIELAB is a uniform Euclidean space and therefore distances between points can be used to represent approximately the perceived magnitude of color differences between object color stimuli viewed under similar conditions. For the sake of simplicity, in the present work the CIE 1976 L, a, b (CIELAB) color difference definition was adopted, see references in [4][2] for details.

b) Using a color space such as CIELAB implies that colors refer to human visual sensibility, and thus it would be preferable to use information about the visual spectral sensibility of the spider. Nevertheless, to the best of our knowledge, such information has not yet been recorded for the jumping spider used in this work, *L. jemineus*. Preliminary studies of other salticid spiders suggest the presence of photopigments with absorption bands in the UV (ca. 360nm) and the green (ca. 520nm) [5]. Since the spectral information available to us through the use of microspectrophotometry is restricted to the visible wavelengths, we limited our analysis to the use of the nomogram proposed by Govardovskii [6] in order to obtain the normalized absorption spectrum of the 𝜶 band of A1 type pigment with 𝜆max=520nm. This curve was used to calculate the spectral component of each reflectance curve by multiplying the value of the reflectance curve at each point by the corresponding value of the normalized absorption as described in a previous work [2]. The comparison of the curves obtained in this way for both the reflectance of the BOB pattern of each genus and the reflectance of the paints, allowed us to hypothesize which mixtures would most resemble the wasp cuticle.

The green spectral component of six blends of black paint and six blends of orange paint were compared with that of the black and orange color, respectively, of BOB-colored *Baryconus* (S1 Fig.). The best matches in terms of similarity to the black and orange colors of actual wasp cuticle were, respectively, our blend #3 (ivory black + Van Dyke brown) and blend #5 (orange + Venetian red). For black, the difference between wasp cuticle and paint blend was less than 4.5 points, whereas for orange it was 13 points. For reference, 2.3 points is the mean threshold for color differentiation by the human eye [7]. Even though the match between the paints and the spectral characteristics of the cuticle was not perfect, this choice of paint was also the closest fit for the rest of genera, with the exception of *Macroteleia*, orange segment, as shown in S1 Fig. Note that in (A) the black line for *Baryconus* completely overlaps that for paint 3.


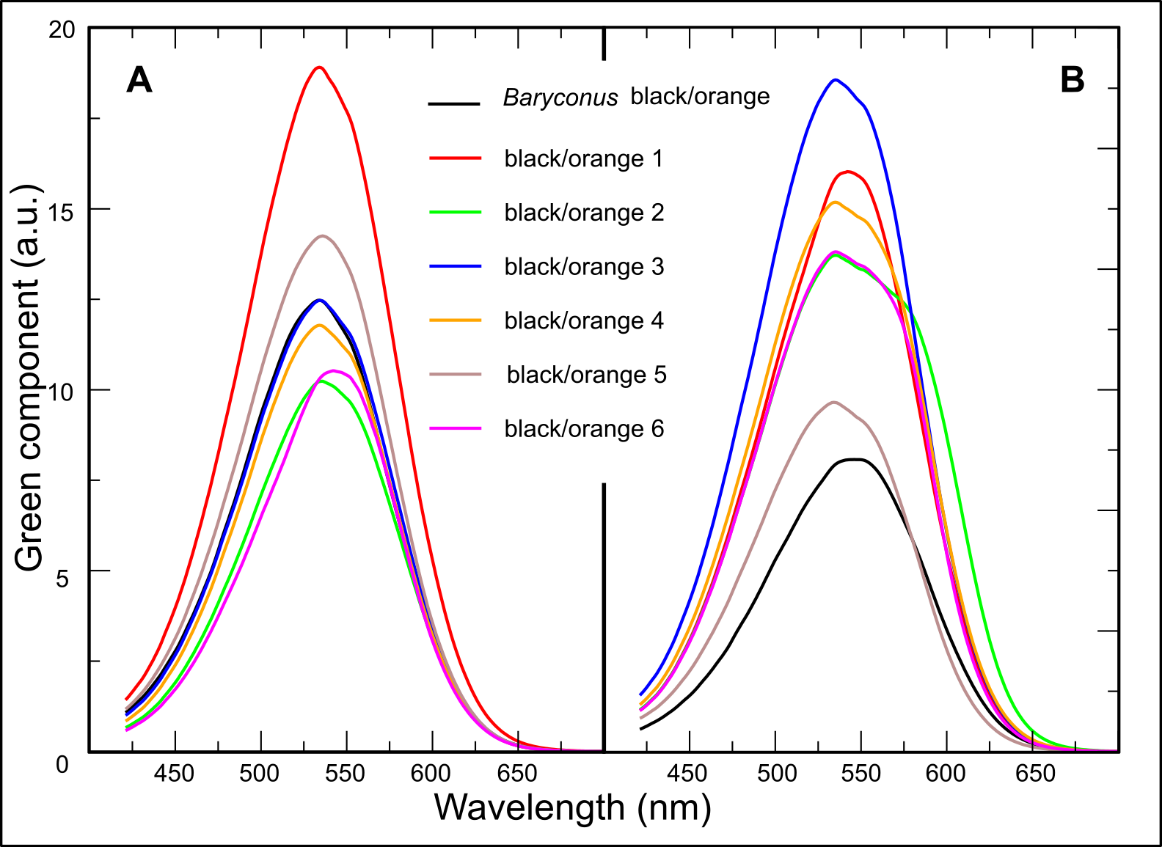


**S1 Fig. Spectral components, in arbitrary units (a.u.), of the reflectance curves.** (A) Black-based blends and (B) orange-based blends of paints compared to black and orange colors in *Baryconus*.


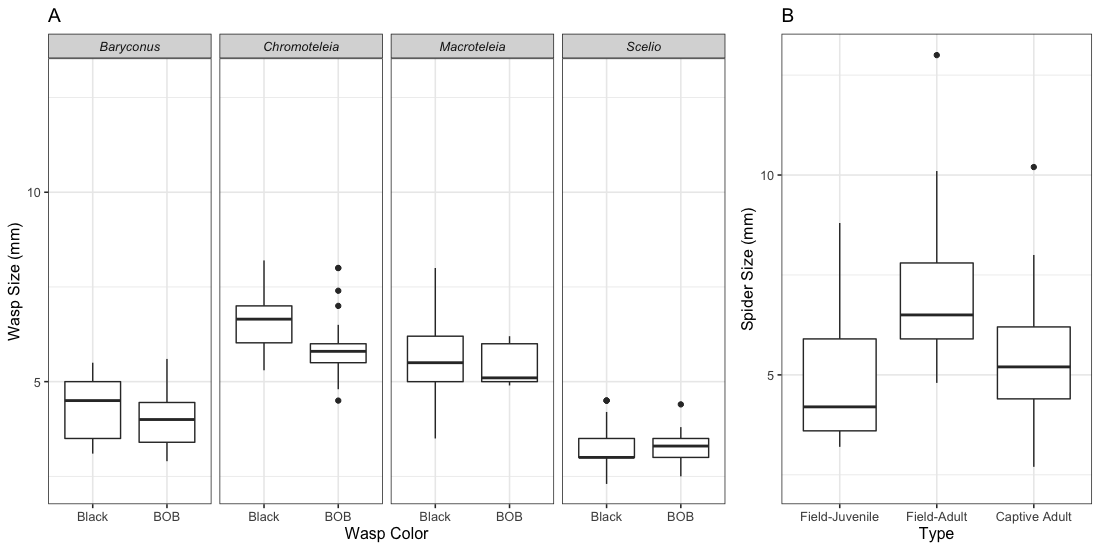


**S2 Fig.**  **Body length of prey and predator used in this study.** (A) Wasps according to their genus and color, (B). spiders according to their origin and ontogenetic stage.

**References**

**1.** Carducci JP, Jakob EM. Rearing environment affects behavior of jumping spiders. Animal Behaviour. 200; **59** (1): 39-46.

**2.** Mora-Castro R, Hernández-Jiménez M, Alfaro-Cordoba M, Avendano E, Hanson-Snortum P. Spectral measure of color variation of black-orange-black (BOB) pattern in small parasitoid wasps (Hymenoptera: Scelionidae), a statistical approach. PLOS ONE. 2019b; 14 (10).

**3**. Brandley N, Johnson M, Johnsen S. Aposematic signals in North American black widows are more conspicuous to predators than to prey. Behavioral Ecology. 2016; 27 (4): 1104–1112.

**4**. Exnerova A, Svadova K, Stys P, Barcalova S, Landova E, Prokopova M, Fuchs R, Socha R. Importance of colour in the reaction of passerine predators to aposematic prey: experiments with mutants of Pyrrhocoris apterus (Heteroptera). Biol J Linn Soc. 2006; 88:143–153

**5.** Kelber A, Vorobyev M, Osorio D. Animal colour vision - Behavioural tests and physiological concepts. Biological Reviews of the Cambridge Philosophical Society. 2003; 78: 81-118.

**6.** Govardovskii VI, Fyhrquist N, Reuter TOM, Kuzmin DG, Donner K. In search of the visual pigment template. Visual Neuroscience. 2000; 17(4): 509-528.

**7.** Fabricant, SA, Smith CL. Is the hibiscus harlequin bug aposematic? The importance of testing multiple predators. 2014; Ecology and Evolution, 4(2): 113-120.
