# Supplementary material for: First evidence for an aposematic function of a very common color pattern in small insects
Source: PLoS One. 2021 Feb 11;16(2):e0237288. doi: 10.1371/journal.pone.0237288 (PMC7877781; doi:10.1371/journal.pone.0237288)
Supplement: Attachment — Submitted filename: Response to reviewers.pdf [file pone.0237288.s002.pdf]

RESPONSE TO REVIEWERS

Academic Editor
PLOS ONE

Dear Phillip Barden,

We would like to thank the reviewers for their critical assessment of our work. In the following response we address each point raised by the reviewers.

General Information

Comments to the Author

In your Methods section, please provide additional information regarding the permits you obtained for the work. Please ensure you have included the full name of the authority that approved the field site access and, if no permits were required, a brief statement explaining why.

Response: We included in the Methods section the information requested.

Please ensure that your manuscript meets PLOS ONE's style requirements, including those for file naming.

Done ✔

1. Is the manuscript technically sound, and does the data support the conclusions?

The manuscript must describe a technically sound piece of scientific research with data that supports the conclusions. Experiments must have been conducted rigorously, with appropriate controls, replication, and sample sizes. The conclusions must be drawn appropriately based on the data presented.

Reviewer #1: Yes

Reviewer #2: Partly

Reviewer #3: Partly

Response: We abide by the suggestions of all the reviewers, which strengthened the content of the article, so we consider that the manuscript is technically sound and the data support the conclusions in a clearer and more robust way in this improved version.

2. Has the statistical analysis been performed appropriately and rigorously?

Reviewer #1: I Don't Know

Reviewer #2: No

Reviewer #3: Yes

Response: To clarify, the questions from all reviewers regarding statistical tests have been answered (please see the details in each of our responses), and in most cases we followed the suggestions of all the reviewers.

3. Have the authors made all data underlying the findings in their manuscript fully available?

The PLOS Data policy requires authors to make all data underlying the findings described in their manuscript fully available without restriction, with rare exception (please refer to the Data Availability Statement in the manuscript PDF file). The data should be provided as part of the manuscript or its supporting information, or deposited to a public repository. For example, in addition to summary statistics, the data points behind means, medians and variance measures should be available. If there are restrictions on publicly sharing data—e.g. participant privacy or use of data from a third party—those must be specified.

Reviewer #1: Yes

Reviewer #2: No

Reviewer #3: Yes

Response: It is not clear to us why Reviewer #2 says no. To clarify, the code and data are available in this repository: https://malfaro2.github.io/Mora_et_al2/. The complete datasets are deposited there, along with the code to read it and to reproduce the statistical analysis that was done in this paper.

4. Is the manuscript presented in an intelligible fashion and written in standard English?

PLOS ONE does not copy or edit accepted manuscripts, so the language in submitted articles must be clear, correct, and unambiguous. Any typographical or grammatical errors should be corrected at revision, so please note any specific errors here.

Reviewer #1: Yes

Reviewer #2: Yes

Reviewer #3: Yes

REVIEWER 1 (chosen color to highlight the suggestions of this reviewer in the manuscript)

This is a novel approach to a complex question, and there is little doubt of the efforts made to obtain these data, even if they are 'noisy' (biology is noisy!). I think that the material is interesting and should be published, but as written the rationale behind certain tests is a bit unclear and the writing could be streamlined so as not to lose the reader. Please note that I have replied "I don't know" about the statistics primarily because I lack the skills to evaluate the colour-based information. The introduction is very well-written and the referencing is very good. The methods are equally thorough! I can imagine, if there is a need to shorten the manuscript, that considerable (and commendable) detail from this section could be removed.

L. 46. Define BOB on first mention

Done ✔ We clarify what the term BOB refers to in the first sentence of the introduction.

Methods

Done ✔ Detailed comments below. The methods are extremely detailed, and yet I am confused, because there is reference that animations were used, but there is no detail on this at all, so in the end, I am forced to think it was using live prey and rice. What is the situation with reference to animations (specifically "Editing was done in Adobe Premiere, the animations and layout in Adobe After Effects")? Also, I suggest the authors look at references by Cynthia Tedore:
https://academic.oup.com/beheco/article/26/2/510/258466
https://jeb.biologists.org/content/216/9/1744.short

The word animation causes confusion and therefore it was removed. In this research we did not use animations, but rather lures, which consisted of painted grains of rice that served as false movable prey. The filming crew used text (arrows) animations to highlight predator movement to help us as researchers analyze the data. The animated videos with arrows were used internally by the co-authors only for data analysis and collection, so the word animations does not apply.

Done ✔ L. 174. Change "in" for "at" 60 fps

Done ✔ L. 174-175. As you have not yet discussed animations, this information comes out of the blue- so far all we have read is about live-prey capture observations. Move to next section? .

The word animation causes confusion therefore it was removed. See paragraph above.

Done ✔ L. 199. This section is very detailed, yet we are unclear of the overview of the test or why it was performed. Before going into details of the spectral details

of the paint used on rice, we need a clear understanding of how the rice, the spider, and the arena 'interacted'. How was the rice made to move? Did it float? Based on the comment in L 175, I half expected it to be an animation of rice. At this point I am getting thoroughly lost. The view of the forest is being lost to the detail of the trees, so to speak. The first thing to do is move any description of the automated cage up, so that the reader can understand the arena before these details are discussed.

Thank you for your comment, indeed the word animation caused confusion and as we mentioned previously it was eliminated. We have also attempted to clarify this part by separating the methodologies for live prey from those for false prey, and by focusing in this paragraph only on the false prey (rice lures). We thoroughly reorganized the content of both methodologies.

Done ✔ L. 256, add comma after "prey"

Done ✔ L. 310. How was the dragline observed? And why?

Since we were observing a predator-prey system not studied before, we wanted to analyze various variables (some more likely to be related to predation, others less so) in order to obtain the greatest amount of information from the data. In the case of silk we decided to leave it as variable since the multinomial model does include it as an important one.

See in manuscript phrases such as:

"Similarly, not using silk is associated with an increased chance of detecting versus attacking ($p = 0.0160$), which makes sense since these spiders use silk only when attacking."

It is important to note that in the lure experiment, the variable silk was not found to be associated with spider behavior.

The dragline variable was considered as noticeable (observed with magnifying glass) when the spider used the dragline in the final jump before attacking the prey, but it was not considered as "present for hunting" when it was only deposited for walking and was less noticeable. We have made this clarification in the text.

Done ✔ L. 323. How did you define "similarly" versus "differently"?

We have clarified this sentence by changing the word "similarly" to the phrase "in the same way" and the word "differently" with the phrase "in a different way". To clarify, the 2 categories we are referring are the following:

1. *L. jemineus* took the same actions with both black and BOB wasps
2. *L. jemineus* took different actions with black and BOB.

Results

Done ✔ It is striking that actually there are very few differences between spider category and wasp pattern colour overall…

Indeed. As stated in one of our hypotheses, we were expecting to see greater differences, but the data did not confirm this, as was stated: "Other factors such as wasp color, wasp size, spider size, and wasp genus did not make a significant difference when included in the full model".

Done ✔ In experiments of spiders with live prey you refer to "captive-adults", which I think belong in the category previously referred to as "reared". Please use expressions consistently.

Thank you for this comment. We have now homogenized the use of these words in figures, tables and text to "captive adults" to facilitate clarity, and we defined it when first mentioned (new sentence in the last paragraph of the introduction)

Done ✔ I am sure Fig 3 could be a supplementary figure, if needed, as could Fig 5 (possibly 8, too?).

We relocated Figures 3 and 5 to the supplementary material.

Done ✔L. 401. Are you sure they only have a dragline when attacking? Many salticids deposit a dragline while walking.

The dragline was considered as noticeable when the spider used the dragline in the final jump before attacking the prey, but it was not considered as "present for hunting" when it was only deposited for walking and was less noticeable. We clarify these aspects in the manuscript.

Done ✔ I am not an expert in colour analysis, but I thought the approach taken in Fig 7 was imaginative and helpful. However, I was a bit lost about the rationale for the bioluminescence testing.

Regarding the use of bioluminesce vs reflectance, we clarify the following:

1. The information compiled in Fig 7 (now figure 6) is related to reflectance, not bioluminescence. The latter was used only in the toxicity analysis.
2. Short explanation about this Figure: the consolidation of the information obtained by using the reflectance and the spectral absorption of a plausible photosensitive pigment for the jumping spider allowed us to estimate whether or not the spider can differentiate between black and orange cuticle of the wasps.

3. More detailed explanation: The amount of light reflected by any object and then absorbed by the eye of a viewer is the maximum amount of information available to be processed and interpreted for example as contrast and color differentiation. As a way of estimating this parameter, we measured the light reflected by black and orange portions of the exoskeleton of the wasps and then multiplied it by the spectral absorption of a pigment with absorption peak in the green. The physiology of the eye of the jumping spider we studied has not yet been fully described in terms of the pigments it contains, but our choice is based on what is known for other salticid spiders [see Kelber et al. cited in the manuscript]. We used the normalized absorption spectrum of the α band of A1 type pigment calculated according to Govardovskii's method. This argumentation is mentioned within the Materials and Methods Section, and currently described in S2 Appendix.
4. In order to emphasize the relationship between reflectance and the contrast parameter proposed in our work, the following line was added in the discussion paragraph that cites this figure: "Also, the green spectral components of the black and orange colors of the BOB pattern from Fig 5, used for the calculation of the absorption contrast, were included as a visual aid."
5. Also, in the Materials and Methods section, the subtitle regarding the use of reflectance of color contrast estimation was renamed to emphasize this procedure.
6. Bioluminescence was employed in this work in the *Vibrio fischeri* ecotoxicological test (Microtox ®). In this test, the bioluminescent bacteria was exposed to dilutions of the wasp extracts, which results in a decrease in the bioluminescence emitted by the bacteria. Consequently, the percentage of this decrease is employed as the test end-point, to determine the EC50 after the evaluation of a dose-response curve.

Discussion

Done ✔ L. 591. You are correct is saying that learning of aposematic signals is often assumed. Nevertheless, this need not be the case (theoretically or in practice), as I have argued at length, demonstrating innate aversion of ants by jumping spiders (and mantises) on numerous occasions:

Nelson, X.J., Jackson, R.R., Li, D., Barrion, A.T., Edwards, G.B., 2006. Innate aversion to ants (Hymenoptera : Formicidae) and ant mimics: experimental findings from mantises (Mantodea). Biological Journal of the Linnean Society 88, 23-32.

Nelson, X.J., Jackson, R.R., 2006. Vision-based innate aversion to ants and ant mimics. Behav Ecol 17, 676-681.

https://link.springer.com/article/10.1007/s10164-020-00639-1

Thank you for this suggestion. We have now expanded the discussion of innate aversion, including references to Nelson et al. 2006, as well as Fabricant & Smith 2014.

REVIEWER 2 (chosen color to highlight the suggestions of this reviewer in the manuscript)

The manuscript entitled "First evidence for an aposematic function of a very common color pattern in small insects" tested whether a pattern coloration (black-orange-black) common in parasitoid wasps functions as an aposematic signal to indicate toxicity to small arthropod predators. I believe it is a novel test of the idea of aposematism (aposematic coloration has been always tested for vertebrate predators, as the authors state), and I found the results very interesting and worth publishing. However, I believe the manuscript could be polished further. In general, I believe the presentation of results could be more clear, and free from phrases that belong to discussion and methods; that some details of the methods could be included as Supplementary Materials; and, that the Discussion could be strengthened by adding more literature.

Specifically, these are my major recommendations:

Done ✔ 1. The abstract should include more results and less details of the methods. The last sentence of the abstract (L-32-34) is uninformative.

Done ✔ 2. L. 81-86. If you list the predictions in the same order than the results, that facilitates reading.

We have now matched the order in which the predictions are mentioned in the introduction with their presentation in the results.
Also, the prediction of a greater contrast (between black and orange) triggering fewer attacks was not explained, we added a statement.

At the end of the introduction, a prediction statement was included: "We had four predictions: - all-black prey would be preferred over prey with the BOB pattern; - experienced predators (collected in the field) would avoid the BOB pattern more than inexperienced adults reared in captivity; - greater perceived contrast between black and orange colors would trigger fewer attacks from the predator, since this would amplify the aposematic BOB pattern; - when comparing wasp species with the BOB color pattern with all black species, the former would show greater toxicity."

Done ✔ 3. Right below the results there is a section called "experiments with live

prey", that is not line-numbered, and that belongs to the Methods; indeed, some parts are repeating information already present in the methods. Fig 3 should be presented for the Methods section.

The paragraph has been deleted from the Results section, and its most relevant parts have been moved to the statistical analysis section (highlighted in green for clarity).

Done ✔ L. 379-385. Again, most of this paragraph should be in Methods.

The paragraph has been deleted from the Results section, and its most relevant parts have been moved to the statistical analysis section (highlighted in green for clarity).

Done ✔ L. 395-407. Here, you reader is dying to know whether black wasps were more attacked than BOB wasps, but that is not straightforward to find in this paragraph. In fact, in one part tell us that the wasp color was not significant, but at the end it says that field adults only attacked black wasps… it is confusing. Also, were all wasp genera combined here?

It is not clear if the statistical model (Fig. 4) is accounting for repeated measures, i.e. including the spider ID as a block or random factor in the model. The descriptive statement about the field adults only attacking black wasps has been deleted, since it is only descriptive. Also, the wasp genus was included as one of the covariates, but we could not find statistical evidence of a difference. To clarify, the model groups the most common action per subject, thus we have only one measure per subject (spider), and we do not need to account for spider ID as a random factor.

Done ✔ L. 408-428. This whole page is missing associated statistical values.

Thank you for your comment. On this page we are describing the behavior of the spiders in the experiment by time slots, but we do not have enough observations to make statistical comparisons between time slots, since there are some categories with very few observations, as noted in the original Figure 5 (now figure 3).

Done ✔ L. 442-443. This result about *Baryconus* spectral components of orange and black makes me want to see the probability of attack vs avoid vs detect separated by genus.

The wasp genus was included as one of the covariates for the multinomial model, but we could not find statistical evidence of a difference, hence we did not report the probability in this case. Here is a contingency table that we did not include in the paper, but which is included in the data and code repository: https://malfaro2.github.io/Mora_et_al2/report2.

	Wasp Genera	

Spider Response	Baryconus	Chromateleia	Macroteleia	Scelio	Total Cases
Attack	7	1	2	4	14
Detect	12	44	20	23	99
Avoid	1	10	4	8	23

Done ✔ L. 448-458. This section is again missing statistical tests. If the absorption contrast is a continuous variable, can you run a model of whether the absorption contrast influences the probability of attacking vs avoiding only for those spiders that detected the wasps?

The analysis done in this part is merely descriptive mainly because the number of observations for each genus are not enough to run a model. Nevertheless, in order to improve this part, we:

1. Estimated an error for the absorption parameter using the standard deviation of the reflectance curves. The following sentences were added:
    a. In the Methods for Absorption contrast parameter for the green spectral components of the BOB pattern, the following explanation was included: "The calculations described here were performed using the reflectance curves reported in reference [2]. The standard deviation of the areas under curve from the green components were used for estimating an error for the calculation of the absorption contrast."
    b. In the results: The standard deviation for the areas under the curve, used for estimating the error in the calculation, are shown as percentages inside square brackets.
2. We rewrote the lines about the analysis of the relationship between the absorption contrast and the behaviors observed. It now reads: "In general, the dispersion of the reflectance data taken from reference [2] prevent us from making strong assertions, but some features or tendencies can be pointed out from the comparison between the contrast absorption parameter and the results of experiments with live prey summarized in Fig 6. As discussed in reference [2] , the dispersion in those measurements derives from several factors, including the fact that for this study, species were grouped into genera. As mentioned previously, the reflectance curves of *Baryconus* have the least dispersion. Therefore the estimated error in the contrast parameter is the lowest between the genera studied. Nevertheless, the contrast parameter resulted in a value that is within the error, meaning that black and orange cuticle segments have the same absorption. This is consistent with the fact that *Baryconus* presented the higher percentage of "attack" events, suggesting that if color perception is the only variable considered, in this case black and orange are not conspicuous from the

point of view of the spider. *Scelio* and *Macroteleia* have higher absorption contrast, and higher orange contribution, suggesting that in those cases the spider should be more likely to differentiate between black and orange. In fact, no experiment involving *Macroteleia* wasps resulted in an "attack" event, all of them fell into the category "detect". The results for *Chromoteleia* are similar in the sense that all the events were distributed between the categories "detect" and "avoid", and the contrast parameter is the second highest in terms of absolute value. Nevertheless, the standard deviation of reflectance measurements is the highest among the genera considered, resulting in an absorption parameter that is within the error estimation. Even though the relationship between the BOB pattern and the behavior of the predator should be taken with caution because of the estimated error in the calculations, we hypothesize that the contrast difference between black and orange from the point of view of the predator visual capabilities is an important factor to be taken into account in behavioral experiments."

Done ✔ Fig 7. The percentage is calculated over which total?

The percentages in the former Fig 7 (now Fig 6) are calculated with respect to:

1. the total trials recorded for each genus in the case of BOB specimens
2. the total trials independent of genus for the black wasps

The reason for this difference is that in a previous work, we concluded that the black color from different genera was spectrally very similar, whereas the orange color showed a tendency to be spectrally different from one genus to another.

A summary of the data collected in experiments with live prey, analyzed without the differences between genera, can be consulted in former Fig 4.

The following explanation was added to the Results section, specifically in the description of results of the Green spectral components of the BOB pattern:

"The observed events, classified in the categories "detect", "avoid" and "attack", as explained within the Methods section, are presented as percentages calculated with respect to total number of trials for each genus in the case of BOB specimens, and the total number of trials for black specimens of all genera. This choice is based on a conclusion of a previous work by our group [2], which suggests that black color is spectrally similar between genera."

Done ✔ Table 1. How is the same and different calculated here?

We have clarified this sentence in L. 323 by changing the word "similarly" to the phrase "in the same way" and the word "differently" with the phrase "in a

different way". To clarify, the 2 categories we are referring are the following:

3. *L. jemineus* took the same actions with both black and BOB wasps
4. *L. jemineus* took different actions with black and BOB.

Done ✔ L. 502-514 is missing stats.

Data and code will be added to the repository, however, despite our considerable effort in collecting the specimens for this and other tests, the number of wasp samples we had limited the amount of extracts and number of repetitions. A considerable quantity of these small wasps would be necessary to fulfill this comment/request and for us it was not possible to obtain such amounts in the field, despite multiple visits. However, ecotoxicological data are shown in Table 2 as the LC50 or EC50 values and their confidence intervals (CI), both determined according to the dose-response patterns by the "drc" package in R software (*Daphnia magna*) or the Microtox ® (*Vibrio fischeri*). This data display is widely accepted in scientific literature for ecotoxicological parameters, where differences are estimated based on CI overlap (see references below, including some from our research group). Moreover, due to the employed dilution approach, the values reported as ">100%" indicate no toxicity in the wasp extracts, in contrast to values below 100%.

References:

-Acosta-Sánchez, A., Soto-Garita, C., Masís-Mora, M., Cambronero-Heinrichs, J. C., & Rodríguez-Rodríguez, C. E. (2020). Impaired pesticide removal and detoxification by biomixtures during the simulated pesticide application cycle of a tropical agricultural system. *Ecotoxicology and Environmental Safety*, *195*, 110460.

-Masís-Mora, M., Lizano-Fallas, V., Tortella, G., Beita-Sandí, W., & Rodríguez-Rodríguez, C. E. (2019). Removal of triazines, triazoles and organophosphates in biomixtures and application of a biopurification system for the treatment of laboratory wastewaters. *Chemosphere*, *233*, 733-743.

-Bérard, A., Dorigo, U., Mercier, I., Becker-van Slooten, K., Grandjean, D., & Leboulanger, C. (2003). Comparison of the ecotoxicological impact of the triazines Irgarol 1051 and atrazine on microalgal cultures and natural microalgal communities in Lake Geneva. *Chemosphere*, 53(8), 935-944.

Done ✔ Table 2. Please consider presenting this information in a figure.

The authors consider that presenting the information contained in Table 2 in a figure would not provide additional information to the manuscript. Moreover, the current Table 2 presents individual EC50 values determined for individual wasps and two benchmark organisms; the potential organization of this data in a figure could be more confusing. The use of figures could be useful to represent the dose-response curves separately employed to determine the EC50 values from each extract and for each bioindicator (*Daphnia magna* and Microtox test). However these plots are not commonly shown, given that they are basically used

for EC50 estimation (they would represent 14 plots in this work); EC50 represents a parameter that summarizes the behaviour of dose-response plots. The display of EC50 (or analogous toxic units, TU) values in the form of a table is a common practice in scientific literature; some references are provided as examples:

-Wollenberger, L., Halling-Sørensen, B., & Kusk, K. O. (2000). Acute and chronic toxicity of veterinary antibiotics to Daphnia magna. *Chemosphere*, *40*(7), 723-730.

-Zhu, X., Zhu, L., Chen, Y., & Tian, S. (2009). Acute toxicities of six manufactured nanomaterial suspensions to Daphnia magna. *Journal of Nanoparticle Research*, *11*(1), 67-75.

-Asghari, S., Johari, S. A., Lee, J. H., Kim, Y. S., Jeon, Y. B., Choi, H. J., ... & Yu, I. J. (2012). Toxicity of various silver nanoparticles compared to silver ions in *Daphnia* magna. *Journal of Nanobiotechnology*, *10*(1), 14.

Masís-Mora, M., Lizano-Fallas, V., Tortella, G., Beita-Sandí, W., & Rodríguez-Rodríguez, C. E. (2019). Removal of triazines, triazoles and organophosphates in biomixtures and application of a biopurification system for the treatment of laboratory wastewaters. *Chemosphere*, *233*, 733-743.

Done ✔ L. 560-567. Also consider that the paint was matching the genus with the least differences between orange and black.

The following sentence was added to the manuscript for clarification: Also, the absorption contrast of the paint chosen is somewhere between the contrast for *Scelio* and *Baryconus*, and might not be enough to trigger a clear response from the spider.

Done ✔ Is there any literature (even in vertebrates) where painted inanimated objects served to test aposematic signals? Perhaps you want to include that.

Thank you for the comment, we included a more detailed explanation of the implementation of false prey to test aposematic signals and additional references.

Done ✔ What happened with the black vs white walls on the arena?

The background was not significantly associated with the spider behavior. For clarity, we added the statistics for each independence test in the following paragraph:

"Table 1 presents the results from the experiment with false prey. Each contingency table was tested for independence, and there was no evidence of dependence for background ($G = 2.0879$, p-value = 0.1485), color ($G = 0.06728$, p-value = 0.7953), or presence of silk ($G = 0.0089311$, p-value = 0.9247). In other words, spider behaviors are not associated with background color (black or white walls in the arena), the color of lure that was detected first, or the presence or

absence of a noticeable silk dragline."

Done ✔ Discussion: please consider including more literature, for example about field vs captivity-reared animals regarding familiarity with aposematic signals.

More references were added and the discussion was strengthened with results from other studies involving naive predators reared in captivity.

Minor comments:

Done ✔L 55-56. Is there any report of jumping spiders preying on parasitoid wasps? If so, please include the reference to support the selection of this spider as a predator.

The reference was included in the introduction.

Done ✔ L. 92. Please provide m.a.s.l for that site.

We added the m.a.s.l for this site (scelionid collection site) and also for salticid collection site.

Done ✔ L. 103-107. You can say here that all four genera have BOB and black specimens, so the reader doesn't have to guess.

Done ✔ L. 115-123. Are the four genera of wasps also found at this locality?

Yes, however we decided to collect them at the other site mentioned in the article because we found more specimens of the four genera in less time, which was crucial due to the large amount of sampling involved in this research. Both sites are in the Central Valley. To clarify this we rewrote the first sentence in Materials and Methods to read: "The collection site with the greatest number of scelionid wasps of interest was in the Central Valley of Costa Rica, in a forest patch …"

Done ✔ L. 133-146. This section could be shortened if you remove some irrelevant details. Also, some details of the "Salticid rearing" section could be moved to Supplemental material.

This section was transferred to Supplemental material.

Done ✔ L. 174 – which animation?

The word animation causes confusion therefore it was removed. In this research we did not use animations, we used lures, painted grains of rice as false movable prey. The filming crew used text (arrows) animations to highlight predator movement to help us as researchers analyze the data. The animated videos with

arrows were used internally by the co-authors only for data analysis and collection, so the word animations does not apply.

Done ✔ L. 176-197: These bullet points can be redacted in a paragraph, and some of the points could be shortened as they are too wordy. For example, point 6 could simply be: Each spider was allowed 60 s of acclimatization in the chamber, before introducing the prey.

All were synthesized and summarized.

Done ✔ L. 212-213. Was the reflectance of the paint measured on painted grains of rice, or on which surface?

On the same surface in which the experimentation with the predator was going to be carried out, that is, on painted grains of rice.

Done ✔ L. 199-249: Many of the details on the selection of the paint should be moved to Supplementary Material.

Moved. Including the former Fig 8, which corresponded to the results of this analysis.

Done ✔ L 315-316. Please explain this section better (frequency according to time). Why do you have 5 min and 10 min time slots mixed?

The time slots have been unified to 10 min, in the text and in Fig 4. For clarity, the following phrase

"Additionally, frequency of actions for each group of spiders were calculated according to time, in order to define a clear timeline of spider behavior in the controlled experiments."

has been corrected to:

"Additionally, the number of individual actions for each group of spiders was calculated for each of the four 10-minute time slots, and distinguished between the wasp colors they were acting on, in order to define a clear timeline of spider behavior in the controlled experiments."

Done ✔ L. 443-447. These sentences are methods.

Indeed, this information was already in the methods. The sentences mentioned by the reviewer were substituted with:

"The absorption contrast parameters, calculated as described in the Methods section..."

Done ✔L. 476-483. This should be considered for methods and as Supplementary Material.

This section was transferred to Supplemental material.

Done ✔ Fig. 8. Can you please explain why the paints were compared to the *Baryconus* only. Considering that you also reported that the black and orange of this genus had similar spectral components, could that influence your results with the lures?

1. We used *Baryconus* because this data set showed low dispersion compared with those for other scelionid genera, as reported in a previous work by our group. This choice was stated in the Materials and Methods section, within the part called "Observations of spiders with false prey in automated cage",
2. When comparing the available pigments with the spectral characteristics of the black and orange segments of the other genera, this choice represents the closest fit for the rest of the genera, with the exception of the orange in *Macroteleia*. This is because the pigments are not an exact match for any of the cuticle reflectance spectra.
3. Going further with the analysis, when calculating the absorption contrast for the pigments, we see that it would be located somewhere between that same parameter for *Baryconus* and *Scelio*. In fact it is closer to *Scelio* than to *Baryconus*.
4. Regarding results of experiments with a lure or false prey, it is possible that the contrast between black and orange paint was not enough to trigger a clear response from the spider. A comment about this point was added in the text.

   In order to support statements 2 and 3, we added the green spectral components of the chosen paint blends together with the green spectral components of the 4 genera studied in former Fig 6. Also, in the Methods section, "Observations of spiders with false prey (rice lures) in the automated cage" was extended as the result of comments from reviewers. There is now a paragraph dedicated to the interaction between spiders, lures (made of painted rice) and the experimental arena (marked in blue in the manuscript), in which the last sentence of item b) reads: "With respect to the four scelionid genera included here, we chose to use the previously reported mean reflectance curves for *Baryconus* because this data set showed low dispersion compared with those for other scelionid genera [2]." Also, in the section about the Choice of oil paint for the lures, which moved to the S2 Appendix, we added the following sentence: "Even though the match between the paints and the spectral characteristics of the cuticle was not perfect, this choice of paint was also the closest fit for the rest of genera, with the exception of *Macroteleia*, orange segment, as shown in S2 Fig. "

Done ✔ L. 503. LC50 should be a concentration that kills 50% of the animals, but it is a percentage, why?

Considering that wasp extracts are complex mixtures that contain many different (unknown) compounds, it is not possible to express the ecotoxicological parameters (e.g. EC50 or LC50) in the units of mg/L (normally employed for pure compounds), given that the effect is the sum of all the potentially toxic compounds in the matrix. In these cases (such as environmental samples for which it is not possible to determine the diversity of compounds they contain), the "pure" sample is considered as the "100%" (extract itself), and subsequent dilutions from this sample are employed in the ecotoxicological tests. Therefore, in the end the parameters are expressed as "the dilution with respect to that 100% that produces the response effect".

EC50 (%) refers to the "effective concentration" that produces 50 % of the response in the ecotoxicological test. In this specific test, it refers to the "relative concentration" of the extract that produces 50% inhibition in the bioluminescence production by *Vibrio fischeri* in the Microtox test. The pure sample (extract) represents the 100%-concentration. Similarly, LC50 (%) represents the "lethal concentration" that produces 50% of lethality in the *Daphnia magna* test; again, the pure sample (extract) represents the 100%-concentration, and the reported value corresponds to a dilution of this sample.

The following lines were added to clarify this point in Materials and Methods:

"LC50 was defined as the relative concentration of the extract (considering the pure extract as 100%) that causes a mortality of 50% in the water fleas."

"...the EC50 value, defined as the relative concentration of the wasp extract (considering the pure extract as 100%) that causes 50% of bioluminescence inhibition."

Done ✔ Table 2: IC? Or CI?

The abbreviation was changed to "CI", i.e. "confidence interval".

REVIEWER 3 (chosen color to highlight the suggestions of this reviewer in the manuscript)

The topic of the manuscript is interesting, as it focuses on a phenomenon described only in fairly large animals, but common among the small arthropods (not only insects). Aposematic function of colour patterns was previously tested in much larger animals (e.g. medium and large insects, reptiles and amphibians). There are three major reasons for that: 1) it is much easier to use larger animals for tests; 2) aposematism was earlier reported from such large animals and 3) if aposematism has evolved in an organism there should be a receiver (predator) the feature is

advertised to. Typically, such predatory animals were assumed to be animals larger than the prey, with high visual capacity – high enough to perceive the signal (vertebrates, typically birds). This is indeed the first study that exploits minute predator with high visual capacity in the context of interactions with BOB-patterned aposematic prey. The model spider seems properly selected (common, easily identifiable predator).

Done ✔ The insects used in the experiments seem, however, less convincing as prey models and therefore they should be described in more detail. Our knowledge about tropical scelionids is still very scanty. Due to the lack of taxonomic knowledge the authors identified the species of wasps from 1 out of 4 genera. This can potentially create some problems in interpretation, as similar species of scelionids may have different behaviour and chemical constitution and although salticids are mostly visual hunters they also exploit other senses in prey capture. It is, therefore, possible that other than visual cues might have been exploited during interactions with different live as well as coloured false prey. I personally admire the workload and time sacrificed to collect the insects in the field. Still the fact that we don't know exactly the actual material as much as we would like to should be critically discussed.

We were aware of this problem, but did not fully explain this in the manuscript. We have therefore added the following sentence at the end of Scelionid collection (Materials and Methods). "While there is a chance that some of the morphospecies included a mixture of more than one species, this problem was minimized (though not totally eliminated) by always collecting from the same site and carefully examining the specimens under a stereomicroscope." In addition, as already mentioned in the manuscript, voucher specimens have been deposited in the Zoology Museum of the University of Costa Rica.

Done ✔ Description of some methods seems too detailed (e.g. concerning salticid and their egg sac collection), while other methods, which seem important to understand and interpret the results, are scanty (e.g. the behaviour of spiders and scelionids in the experimental chambers, motion pattern of false prey).

Thank you for this comment, which was also mentioned by other reviewers. We have separated the methodologies for live prey and false prey, and thoroughly reorganized the content of the two methodologies.

Done ✔ In the results there seem to be statements without statistical support (and a name of a test) e.g. lines 420-428, 451-453 referring to Fig. 7. Again in lines 490-492 referring to Table 1 there is information that the data were "…tested for independence, and there was no evidence of dependence in any 492 of the cases", but no results and the tests used were given. Similarly in the discussion

(lines 532-535) conclusions are drawn without a reference to a statistical test. Appropriate tests should be carried out or shown to check the statements.

The clarifications referring to Fig 7 were added according to a comment from Reviewer 2 about L. 448-458, see above. An error estimation was included for the calculation of the absorption contrast, and changes in the Methods, Results and Discussion were made accordingly. The modifications appear in green and not in red (color corresponding to this reviewer) because we addressed the comments of Reviewer #2 before those of Reviewer #3.

The paragraph describing Table 1 and the Table have been corrected to account for suggestions from reviewers. This paragraph:

"Table 1 presents the results from the experiment with false prey. Each contingency table was tested for independence, and there was no evidence of dependence in any of the cases (A, G. In other words, spider behaviors are not associated with background color, the color of lure that was detected first, the use or non-use of silk, or predator size."

was changed to:

"Table 1 presents the results from the experiment with false prey. Each contingency table was tested for independence, and there was no evidence of dependence for background ($G = 2.0879$, p-value = 0.1485), color ($G = 0.06728$, p-value = 0.7953), or presence of silk ($G = 0.0089311$, p-value = 0.9247). In other words, spider behaviors are not associated with background color (black or white walls in the arena), the color of lure that was detected first, or the presence or absence of a noticeable silk dragline."

Also, we added percentages of the total to the counts in Table 1, to make it easier for the reader.

In lines 532-535 the phrase: "on five occasions field-captured *Lyssomanes* spiders consumed black wasps (all belonging to the genus *Scelio*)" was eliminated.

Done ✔ In the study "the use of silk" by the spiders was included in the analyses (e.g. Table 1, lines 209, 310). It is not explained, however, why just this variable was included. In lines 401-402 it is also mentioned that "these spiders use silk only when attacking". This seems unlikely for a vegetation-dwelling spider, which typically should exploit draglines in locomotion and jumps (including jumps onto prey).

Since we were observing a novel predator-prey system, we wanted to analyze various variables such as silk in order to obtain the greatest amount of information from the data, and including this turned out to be worthwhile.

See in manuscript phrases such as:

“Similarly, not producing a noticeable silk dragline is associated with an increased chance of detecting versus attacking ($p = 0.0160$), which makes sense since these spiders produce more silk when attacking.”

This clarification about silk use is an important one. We have clarified throughout the manuscript that what we were recording was the presence of noticeable amounts of silk (while jumping toward the prey), and “absence of silk” has been changed to read “absence of noticeable silk”. Thank you for drawing this to our attention.
