# Supplementary material for: First evidence for an aposematic function of a very common color pattern in small insects
Source: PLoS One. 2021 Feb 11;16(2):e0237288. doi: 10.1371/journal.pone.0237288 (PMC7877781; doi:10.1371/journal.pone.0237288)
Supplement: Attachment — Submitted filename: Response to reviewers.pdf [file pone.0237288.s003.pdf]

Response to reviewers (corrections will be indicated in "the manuscript with track changes" in red)

Rebuttal letter

PONE-D-20-22240R1

First evidence for an aposematic function of a very common color pattern in small insects.

PLOS ONE

Academic Editor
PLOS ONE

Dear Phillip Barden,
We would like to thank the reviewers for their critical assessment of our work. In the following document we address each point raised by the reviewers.

Comments to the Author

1. If the authors have adequately addressed your comments raised in a previous round of review and you feel that this manuscript is now acceptable for publication, you may indicate that here to bypass the "Comments to the Author" section, enter your conflict of interest statement in the "Confidential to Editor" section, and submit your "Accept" recommendation.

Reviewer #1:(No Response)

---

2. Is the manuscript technically sound, and do the data support the conclusions? The manuscript must describe a technically sound piece of scientific research with data that supports the conclusions. Experiments must have been conducted rigorously, with appropriate controls, replication, and sample sizes. The conclusions must be drawn appropriately based on the data presented.

Reviewer #1: Partly

Done ✔ Response: *We abide by the suggestions of all the reviewers, which strengthened the content of the article, so we consider that the manuscript is technically sound and the data support the conclusions in a clearer and more robust way in this improved version.*

---

3. Has the statistical analysis been performed appropriately and rigorously?

Reviewer #1: I Don't Know

Done ✔ Response: *We added an introductory paragraph for clarification in the methods section:*

"There were three different statistical analyses in this work: (1) an experimental design with spiders and live prey, that uses a multinomial model to describe 136 trials, with each trial as the statistical unit, and the most common spider activity per trial as a response (2) a statistical description of the timeline for the same trials, taking each spider action as the statistical unit, and (3) an experimental design with spiders and false prey in the automated cage, with 30 separate trials."

We abide by the suggestions of all the reviewers, which strengthened the content of the article, so we consider that the statistical analysis been performed appropriately and rigorously in a clearer and more robust way in this improved version.

---

4. Have the authors made all data underlying the findings in their manuscript fully available? The PLOS Data policy requires authors to make all data underlying the findings described in their manuscript fully available without restriction, with rare exception (please refer to the Data Availability Statement in the manuscript PDF file). The data should be provided as part of the manuscript or its supporting information, or deposited to a public repository. For example, in addition to summary statistics, the data points behind means, medians and variance measures should be available. If there are restrictions on publicly sharing data—e.g. participant privacy or use of data from a third party—those must be specified.

Reviewer #1: Yes Done ✔

---

5. Is the manuscript presented in an intelligible fashion and written in standard English? PLOS ONE does not copyedit accepted manuscripts, so the language in submitted articles

must be clear, correct, and unambiguous. Any typographical or grammatical errors should be corrected at revision, so please note any specific errors here.

Reviewer #1: Yes Done ✔

Major comments reviewers 1 and 2

Reviewer 2:

Comment 1: Figs 3 and 4 are very difficult to interpret and should be heavily revised. I, along with Reviewer 1, am having a very difficult time understanding where these data come from and what they are suggesting relative to the authors' conclusions – particularly Figure 3. The two figures also appear to be contradictory. An additional concern is that there are no units on the axes and that Figure 3 caption is very brief. Add units to the axes in both figures if they are to be kept in their current form and otherwise revise to make the results clearer as suggested by Reviewer 1.

Done ✔

Response: See response to comment 1 for reviewer 1 *

Comment 2: Fig 4, why is this a stacked bar graph? This makes it much more difficult to directly compare BOB and Black wasp categories. In addition, I recommend including a label for the "detect" "attack" and "avoid" panels, as well as noting how the observations were binned in this scheme in the figure caption.

Done ✔

Response: See response to comment 1 for reviewer 1 *

Comment 3: Line 495: "no spider consumed a wasp with BOB coloration." This appears to be among the more compelling evidence for aposematism, yet results are not presented except when combined with another "attack" behavior. Could these data be presented to support this sentence in the discussion?

Done ✔

Response: We have added this Table in the Appendix:

S4 Table: Frequency of individual behavioral responses experienced by *L. jemineus* when confronted with live wasps, with seven ungrouped behaviors, per wasp color. The total individual actions are n = 954.

	Black	BOB	TOTAL
1.Alert and swivel	160	192	352
2.Follow or stalk	99	111	210
3.Crouch, jump and contact prey	99	101	200
4.Pierce and ingest	8	0	8
5.Prey undetected or ignored	83	48	131
6.Detect visually and withdraw	14	37	51
7.None	2	0	2
TOTAL	465	489	954

And this paragraph has been changed in Results:

When the behavior of all spiders is analyzed individually and during the 40 min and in the different time slots a greater complexity of behaviors is observed, specifically in that the field adults usually differed from the captive adults. Figure 4 summarizes the 954 actions taken by all spiders throughout the timeline of the experiment, and Table S4 shows the detailed count with the original individual actions.

Comment 4: The toxicity results appear to be the most indicative of aposematism, however, the behavioral data, at least as presented here, are not conclusive (among field adults, black wasps are avoided more frequently, BOB wasps attacked more frequently in most time frames according to Fig 4). Given this, I do not feel the final sentence of the abstract "By combining the results from the three types of experiments, together with a statistical analysis, we confirmed that the BOB color pattern plays an aposematic role." is warranted. Please revise this conclusion to be more equivocal (e.g. "some results suggest"), given that the data do not definitively support this conclusion.

Done ✔

Response: Last sentence of abstract now reads: "At least some of the results suggest that the BOB color pattern plays an aposematic role."

Comment 5: L. 298, I still think "in the same way" and "in a different way" is pretty vague and no better than the previous "similarly" and "differently"; some description of precisely what is meant here is really necessary, in my view.

Done ✔

Response: We have changed...

"The response variable in this case was constructed by registering whether *L. jemineus* responded in the same way to black and BOB lures coded as 1, and whether *L. jemineus* responded in a different way to black and BOB lures coded as 2."

To...

"The response variable in this case was constructed by registering whether *L. jemineus* responded in the same way to black and BOB lures, i.e. if it attacked, detected or avoided both the black and BOB lures, coded as 1, and whether *L. jemineus* responded in a different way to black and BOB, i.e. if it attacked black and detected the BOB, or it detected the BOB and avoided the black lures coded as 2."

Reviewer 1:

Comment 1: L. 544-546. The relationship between figs 3 and 4 needs clarification (in the results), as some of the results and discussion appear somewhat contradictory. For example, in the discussion, you state "Moreover, field-collected adults were more likely to detect 544 or avoid than to attack, compared to captive adults which appeared to exhibit a more limited behavioral scale with attack being the most frequent". However, a quick look at Fig 4, which uses the same axes for each row of panels, suggests differently. In part this is because it is expressed in real numbers, not as percentages. However, fig. 3 suggests that field adults NEVER attacked orange wasps, yet fig 4 strongly suggests otherwise. I think these data are from the same experiment yet partitioned differently. Consequently, I am confused about what the results actually were. If I am mistaken in my interpretation, then the explanation for these discrepancies needs to be made crystal in the results and discussion. Alternatively, there is some problem with the classification of the data?

Done ✔

***Response: We added an introductory paragraph for clarification in the methods section and some clarifications (presented in red):**

“There were three different statistical analyses in this work: (1) an experimental design with spiders and live prey, that uses a multinomial model to describe 136 trials, with each trial as the statistical unit, and the most common spider activity per trial as a response (2) a statistical description of the timeline for the same trials, taking each spider action as the statistical unit, and (3) an experimental design with spiders and false prey in the automated cage, with 30 separate trials.”

Additionally, a second analysis was carried out with the same trial, where the number of individual actions for each spider was recorded during each of the four 10-minute time slots, and distinguished between the wasp colors they were acting on, in order to define a clear timeline of spider behavior in the controlled experiments. In this case we had 954 individual actions included in the 136 trials.”

A third analysis of a separate experiment with lures in the automated cage included 30 trials, during which the following variables were recorded: presence of noticeable silk/absence of noticeable silk, spider size, background experimental arena color (black or white) and color of false prey (BOB vs black) that first attracted the spider.

The response variable in this case was constructed by registering whether *L. jemineus* responded in the same way to black and BOB lures, i.e. if it attacked, detected or avoided both the black and BOB lures, coded as 1, and whether *L. jemineus* responded in a different way to black and BOB, i.e. if it attacked black and lured the BOB, or it detected the BOB and avoided the black lures, coded as 2.

Fig 3 and Fig 4 have been changed. Fig 3 is now presented in counts and non-stacked bars, with clearly labeled axis. A label for y axis has been added to Fig 4, the caption has been changed, and the bars have been plotted separately per wasp color, as suggested by Reviewer 2. Also, these clarifications have been added to the results:

“A summary of the most common action per each of the 136 trials included in the final multinomial model is presented in Fig 3. The number of spiders differs between groups, which is why the bars for captive adults are smaller than the ones for field adults and field juveniles.”

And the following paragraph has been erased...

“being a field adult is a significant factor for increasing the odds of detecting versus attacking (coefficient = 2.2727, S.E = 0.8922, p-value = 0.0108), and for avoiding versus attacking (coefficient = 2.6374, S.E = 1.1240, p-value = 0.0189). This shows that field adults are more likely to detect or avoid than to attack, compared to captive adults, which appear to prefer attacking. Similarly,”

...since we agreed with the reviewer that the interpretation of the response “most common action” as opposed to “individual actions” is not clear and can be misleading in this case. Besides, and in order to be consistent, we eliminated from the discussion the following paragraph:

“Moreover, according to the multinomial model field-collected adults were more likely to detect or avoid than to attack, compared to captive adults which appeared to exhibit a more limited behavioral scale with attack being more likely than detect and avoid the most frequent. Thus, adults from the field, which have had more experience, appeared more selective. This is expected, given the complexity of factors and different prey found in natural conditions compared to our artificial breeding environment where the diet consisted of only two types of prey.”

Comment 2: L. 60-63. Technically, whether a spider is di, tri or tetrachromatic is not the cause of high spatial acuity; this is due to photoreceptors in the retina being packed densely in arrays that function as light guides. Perhaps re-work carefully, as colour vision does not equal high acuity, and the causes behind each are different.

Done ✔

Response: We have changed...

“These characteristics have allowed salticids to achieve the highest visual acuity thus far measured in any arthropod, with the ability to classify prey into categories, even if they rely solely on vision [8][9][10].”

To...

“Additionally, jumping spiders visual system exhibits the following pairs of eyes: anterior median eyes (spatial acuity and depth of field), posterior lateral eyes (lower spatial acuity and wide field of view), anterior lateral eyes (support high-resolution spatial vision caused by dense regular patterns of receptors with forward-facing acute zones) and side-facing posterior median eyes (reduced in most genera). All these previous modules of salticids visual system have allowed them to achieve the highest visual acuity thus far measured in any arthropod, with the ability to classify prey into categories, even if they rely solely on vision [8][9][10].”

Minor comments reviewers 1 and 2

line 245: should be "among" instead of "between" as there are more than two genera

Done ✔

line 257: "criterium" should be "criterion" Done ✔

L. 83-89. Please number your predictions Done ✔

L. 181, add comma after “used” Done ✔

L. 186, add comma after “arena” Done ✔

L. 187, add comma after parenthesis Done ✔

L. 273, add comma after “out” Done ✔

L. 370. Can you provide a reference for “spiders produce more silk when attacking”; this is news to me. Done ✔

This is based on personal observation by one of the authors, so we have modified the last part of this sentence to read: "in many cases silk was more evident before attacking the prey (R. Mora-Castro, unpublished), an observation that warrants further research."

L. 418, remove “a” before “previous” Done ✔
