# Supplementary material for: First evidence for an aposematic function of a very common color pattern in small insects
Source: PLoS One. 2021 Feb 11;16(2):e0237288. doi: 10.1371/journal.pone.0237288 (PMC7877781; doi:10.1371/journal.pone.0237288)
Supplement: Attachment — Submitted filename: Response to reviewers.pdf [file pone.0237288.s004.pdf]

January 12, 2021

PONE-D-20-22240R2

First evidence for an aposematic function of a very common color pattern in small insects.

PLOS ONE

Response to reviewers (corrections and additions will be indicated in "the manuscript with track changes" in **red**)

Academic Editor
PLOS ONE
Dear Phillip Barden,
We would like to thank the reviewers for their critical assessment of our work. In the following document we address each point raised by the reviewers.

Comments to the Author

1. If the authors have adequately addressed your comments raised in a previous round of review and you feel that this manuscript is now acceptable for publication, you may indicate that here to bypass the “Comments to the Author” section, enter your conflict-of-interest statement in the “Confidential to Editor” section, and submit your "Accept" recommendation. Reviewer #1: All comments have been addressed

2. Is the manuscript technically sound, and do the data support the conclusions? The manuscript must describe a technically sound piece of scientific research with data that supports the conclusions. Experiments must have been conducted rigorously, with appropriate controls, replication, and sample sizes. The conclusions must be drawn appropriately based on the data presented. Reviewer #1: Yes

3. Has the statistical analysis been performed appropriately and rigorously? Reviewer #1: Yes

4. Have the authors made all data underlying the findings in their manuscript fully available? The PLOS Data policy requires authors to make all data underlying the findings described in their manuscript fully available without restriction, with rare exception (please refer to the Data Availability Statement in the manuscript PDF file). The data should be provided as part of the manuscript or its supporting information, or deposited to a public repository. For example, in addition to summary statistics, the data points behind means, medians and variance measures should be available. If there are restrictions on publicly sharing data—e.g. participant privacy or use of data from a third party—those must be specified. Reviewer #1: Yes

5. Is the manuscript presented in an intelligible fashion and written in standard English? PLOS ONE does not copyedit accepted manuscripts, so the language in submitted articles must be clear, correct, and unambiguous. Any typographical or grammatical errors should be corrected at revision, so please note any specific errors here. Reviewer #1: Yes

Reviewer #1

1) This is a much-improved manuscript and I only have minor revisions suggested. However, I still think the final conclusion (L. 620) should be a bit more circumspect.

Response: We have modified this sentence to read as follows: "To the best of our knowledge, the present study provides the first evidence that this common color pattern might have an aposematic function in these small insects, although further research is needed to confirm this." We have also modified the last sentence of the abstract.

2) Given your results, and I also think the discussion could use a few more sentences to give it more depth. Specifically, you talk about innate aversion in chicken and mantises, yet do not mention work on jumping spiders:

Nelson, X.J., Aguilar-Arguello, S., Jackson, R.R., 2020. Widespread army ant aversion among East African jumping spiders (Salticidae). Journal of Ethology 38, 185-194.

Nelson, X.J., Jackson, R.R., 2006. Vision-based innate aversion to ants and ant mimics. Behav Ecol 17, 676-681.

Additionally, there is relevant research on colour aversion in jumping spiders which should also be added to your discussion:

https://onlinelibrary.wiley.com/doi/full/10.1111/eth.12859

Taylor, L. A., Maier, E. B., Byrne, K. J., Amin, Z., & Morehouse, N. I. (2014). Colour use by tiny predators: Jumping spiders show colour biases during foraging. Animal Behaviour, 90, 149–157. https://doi.org/10.1016/j.anbehav.2014.01.025

Response: Thank you for bringing these papers to our attention. The discussion was strengthened by citing these articles recommended by the reviewer, as well as a couple others.

Other comments

Graphs 3 and 4. The double width bars when there are no 'black' data are a bit confusing. Make the orange bar the same width as the others, and simply not have a 'black bar' where there are no data. I think Table S4 is definitely worth putting in the body of the text. To me, this partitioning makes more sense than the ones used in the graph, and shows things more clearly. In summary: I encourage you to include Table S4 in the main text and revise your bar graphs to remove the "full width" bars when one category is absent.

Response: The suggested corrections have been applied to Fig 3 and Fig 4. Table S4 has been moved to the main text as Table 1, and the subsequent Tables have been renumbered.
